# Vicarious Learning: How Entrepreneurs Enhance a Firm’s International Competitiveness Through Learning From Interlocking Director Network Partners

**DOI:** 10.3389/fpsyg.2020.00689

**Published:** 2020-04-21

**Authors:** Zaiyang Xie, Runhui Lin, Jie Wang, Weiwei Hu, Ling Miao

**Affiliations:** ^1^China Academy of Corporate Governance, Nankai University, Tianjin, China; ^2^Business School, Nankai University, Tianjin, China; ^3^School of Management, Zhejiang Gongshang University, Hangzhou, China; ^4^School of Shangmaoliutong, Zhejiang Technical Institute of Economics, Hangzhou, China; ^5^School of Hotel and Tourism Management, Hong Kong Polytechnic University, Hung Hom, Hong Kong

**Keywords:** entrepreneur psychology, vicarious learning, interlocking director network, cross-border acquisition, learning process

## Abstract

Applying the lens of entrepreneurial psychology, this paper examines vicarious learning as an approach that entrepreneurs can use to overcome external uncertainty of overseas investments by accumulating international know-how and experience through interlocking director connections with other experienced companies. Through the analysis of a sample of Chinese companies, our findings suggest that entrepreneurs obtaining foreign experience from interlocking partners can significantly promote their firm’s international growth when investing in the same country, and that this positive effect is significant in relation to both first-degree and second-degree connections. We further find that, if an entrepreneur makes a connection with an interlocking partner in the same industry, it enhances their knowledge absorption, thereby providing a positive moderating effect, while investing in a country with a strong degree of openness weakens the effect of knowledge application, and thus plays a negative moderating role. This study makes practical and theoretical contributions by exploring specific vicarious learning means for entrepreneurs to enhance their firm’s international competitiveness, and also identifying three different learning processes.

## Introduction

Increasing numbers of entrepreneurs in emerging markets are starting companies through which they are able to make significant contributions to their country’s regional and social economic growth. However, alongside this positive role of entrepreneurship, there exists a great deal of pressure and there are many uncertainties to be faced when competing for a higher market position (Frese and Gielnik, 2014). Specifically, when considering cross-border acquisitions (CBAs) for overseas expansion (Autio and Sapienza, 2000; Yli-Renko et al., 2002; Andersson and Wictor, 2003), entrepreneurs can face challenges in responding to unanticipated risks concerning other countries and in adapting to new institutional environments (Peng, 2005; Luo and Tung, 2007). Given that most new ventures will have limited international experience and correspondingly less capability for doing business overseas, the question of how these entrepreneurs can overcome external uncertainties and deal with mental stress to improve their firm’s international competitiveness is important, and yet it has received relatively little attention in the extant literature (Luo et al., 2011).

From the learning perspective, researchers have contended that learning from others— “vicarious learning”—is one of the most effective ways for firms acquiring and accumulating knowledge to deal with external uncertainty. Under this approach, a firm obtains knowledge by observing the behaviors, actions, and results of the learning objects, and this acquired knowledge then influences the behavioral patterns, decision-making, and performance results of the organization itself (Miner and Haunschild, 1995; Myers, 2018). For firms with less experience, vicarious learning is a more effective and reliable way to accelerate the accumulation of knowledge than a lengthy process of self-experience learning (Huber, 1991; Nathan and Kovoor-Misra, 2002). Moreover, it enables a firm to explore ways of performing tasks and executing their strategies without incurring additional costs or risks (Miner and Haunschild, 1995). From the psychological standpoint, observing and imitating others’ internationalization behavior and strategy can not only improve an entrepreneur’s own decision-making processes but also mitigate their psychological stress and enhance their capability and confidence to cope with difficulties in foreign investment (Bandura and Walters, 1963; Xie and Li, 2017). However, there is little discussion in prior psychology studies of entrepreneurs improving their firm’s international competitiveness through vicarious learning, and even less consideration of the specific ways in which these entrepreneurs might learn from others. To address these gaps in the existing research, our study investigates an approach to vicarious learning in which the entrepreneur builds up an interlocking director network with experienced companies.

Network theory posits that information, knowledge, and resources can be exchanged and transferred by the network (Haunschild and Beckman, 1998; Nam and An, 2018; Xiao et al., 2019). Accordingly, entrepreneurs who establish connections with experienced interlocking partners can benefit in two main ways: (1) by observing partners’ internationalization management practices and improving their own success rate by implementing the same strategic behavior (Burt, 1987); and (2) by better understanding the environment of a specific foreign market and reducing the trial-and-error costs of investments in the same country. Such networking benefits are even more important for a firm with little or no international experience, as entrepreneurs learning from interlocking partners can accelerate their international knowledge accumulation in a more reliable way without any additional costs, in turn helping to decrease their psychological stress and better promote their firm’s internationalization and growth.

**FIGURE 1:**
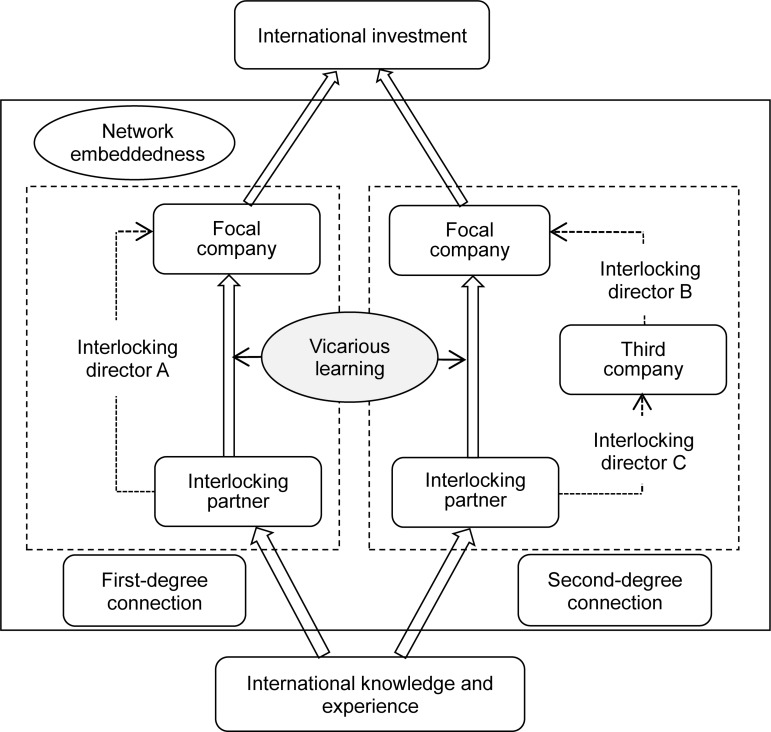
Framework of vicarious learning through two interlocking connections.

For a finer-grained understanding of learning based on an interlocking director network, we propose to investigate two types of network connections: first-degree connections and second-degree connections (Cai and Sevilir, 2010). In a first-degree connection, the focal firm forms a relationship with another partner company through a common interlocking director (A); in a second-degree connection, the focal firm forms an indirect relationship with another partner company through different interlocking directors (B and C) who jointly take positions in a third-party company, as depicted in Figure 1. These two types of connections can bring about different learning outcomes for a firm acquiring international knowledge from interlocking partners. The former enables the firm to observe another company’s management practices more directly and increases the information transfer efficiency, while learning based on a second-degree connection involves the firm acquiring knowledge indirectly, though it can facilitate access to a greater amount of information.

In addition, within vicarious learning, the externally acquired knowledge needs to be effectively absorbed and applied in order to play a role in the firm’s management practices. “Knowledge absorption” comprises a sequential process of an individual understanding, digesting, and absorbing the knowledge learned from other objects, while “knowledge application” is the extent to which the external knowledge then influences the firm’s decision-making and its effect when applied to organizational practices. Different capabilities with regards to absorption and application will moderate the role of external knowledge on a firm’s international strategy. Accordingly, our paper primarily explores how an entrepreneur can improve their firm’s international competitiveness through vicarious learning based on an interlocking director network; specifically, through the two approaches to learning via first-degree connections and second-degree connections. We also examine the ways in which knowledge absorption and application play moderating roles in this relationship.

Overall, our paper makes several contributions to the current research. First, we provide new insights for entrepreneurs looking to overcome uncertainties and reduce psychological stress through learning from experienced interlocking partners. Previous studies have paid little attention to vicarious learning as a means for entrepreneurs with less experience to enhance their firm’s competitiveness, and we fill this gap and propose a more reliable and effective learning approach. Second, we move beyond the existing research in our elucidation of vicarious learning, and, by differentiating the interlocking director network into first-degree and second-degree connections, we are able to provide novel empirical evidence of their different influences and respective roles in a firm’s vicarious learning outcome. Last, we contribute to the learning research by specifying three stages of the vicarious learning process (Myers, 2018), and explore the moderating effects of knowledge absorption and knowledge application in particular, thereby advancing analysis of the organizational learning mechanism.

## Theoretical Background

Modern economic developments have encouraged increasing numbers of entrepreneurs to start up a business in recent years (Kantis et al., 2002; Obschonka et al., 2018). At the same time, these entrepreneurs often face high competition and pressure from the external environment. The issue of helping entrepreneurs reduce mental stress and cope with external uncertainty has attracted much scholarly attention (Gorgievski and Stephan, 2016). Yet there remains a dearth of literature about entrepreneurs’ actions in relation to foreign investments or how they might overcome the difficulties in order to promote their firms’ international development.

In fact, the psychology of entrepreneurs is more important than ever in the context of international investments. With the rapid development of globalization, more and more companies are eyeing overseas markets and making foreign investments to acquire advanced technologies and resources, especially in the form of CBAs (Boateng et al., 2008; Buckley et al., 2009). A CBA is a high-risk investment characterized by high uncertainty and unpredictable outcomes; if it fails, it may bring about substantial costs or even result in stigmatization, damaging the firm’s reputation and credibility (Luo, 2005). Most new ventures possessing limited international experience often find it challenging to deal with external legitimacy or to adapt to new institutional circumstances (Guo et al., 2017). The majority of companies that fail to complete a cross-border transaction lack experience or capability in international businesses. However, CBAs have become a primary way for entrepreneurs to expand their overseas market (Kumar et al., 2019), thus helping them overcome the uncertainties and enhance their firms’ international competitiveness is an urgent and important issue in this field.

Thus, the accumulation of international know-how and experience is crucial for firms undertaking CBAs. Scholars have argued that firms with extensive international experience are more likely to develop the capability of doing businesses overseas and to perform better than those with little or no international experience (Kusewitt and Finkelstein, 1999; Hayward, 2002). Prior studies have identified two approaches to learning for firms wishing to accumulate knowledge: (1) learn from their own experience (experiential learning), and (2) learn from others’ experiences (vicarious learning) (Haunschild, 1993; Barkema and Schijven, 2008; Kump et al., 2015; Lord, 2015; Qian et al., 2018).

From the learning psychology perspective, vicarious learning stands out as an effective way for entrepreneurs to quickly obtain and accumulate international experience, and to do so faster than through their own experiential learning (Haleblian and Finkelstein, 1999; Baum et al., 2000). New ventures are typically latecomers in a “catch-up game” in the global market (Kumaraswamy et al., 2012; Cui et al., 2013), and vicarious learning provides them with a reliable and effective way to improve their market competitiveness without any costs or risk.

Existing literature on vicarious learning and firm acquisitions has determined that companies can learn from the experiences of their network partners to facilitate acquisitions (Haunschild, 1994; Beckman and Haunschild, 2002). Lu (2002) posited that acquirers learn more about advantageous strategic behaviors from other companies in the early stages, and then reduce such vicarious learning with the accumulation of their own experience. By observing and imitating the acquisition activities of their counterparts, a firm can not only garner the necessary acquisition knowledge but also use their acquisition experience (both failures and successes) to make better decisions and enhance the likelihood of completing an international acquisition themselves (Xie and Li, 2017).

To advance vicarious learning research within the domain of entrepreneurship psychology, the present study focuses on the way in which entrepreneurs can learn from other companies by building up an interlocking directors’ network. An “interlocking director” is one who concurrently serves on multiple boards of different companies (Heracleous and Murray, 2001; Westphal et al., 2001). Interlocking directors are widespread and common in companies, and, compared to obtaining information from social media or consultancies, they make it possible for a firm to access the interlocking partner’s board and to acquire more reliable and complete information about their experiences of investing in foreign markets (Tuschke et al., 2014; Zona et al., 2018). Relatedly, Xia et al. (2017) observed that the number of CBAs conducted by an acquirer in a country is positively related to the number of CBAs that its interlocking partner has had in that country. Therefore, this paper explores how an entrepreneur promotes their firm’s international competitiveness—especially with respect to completing a CBA—through vicarious learning from their interlocking partners.

Additionally, the process of vicarious learning has been shown to encompass three stages: knowledge acquisition, knowledge absorption, and knowledge application. Prior studies, though, have tended to focus more on only one of these stages, or have discussed vicarious learning in a general way (Baum et al., 2000; Myers, 2018). We emphasize that firms’ different capabilities with regards to knowledge absorption and application have an influence on the role of the acquired knowledge in the firms’ strategies. This paper further examines the contingency conditions from the perspective of knowledge absorption and knowledge application; specifically, in terms of industry relatedness and the target country’s degree of openness. In sum, our study offers a new theoretical perspective for psychology research regarding entrepreneurs dealing with uncertainties and how they can effectively improve their firm’s competitiveness.

## Hypotheses Development

### Knowledge Acquisition: Vicarious Learning From an Interlocking Partner

A CBA is a highly uncertain investment comprising complicated procedures and requiring a large amount of related knowledge. Entrepreneurs typically have less experience of international investment in their early years and may find it difficult to overcome stress in responding to external uncertainties, but vicarious learning can be an effective way to accelerate knowledge accumulation and reduce their psychological stress in order to promote their firms’ international growth (Francis et al., 2014).

An interlocking company represents a “conduit of information” (Useem, 1984) that could provide knowledge about how to manage country-specific acquisition issues and deal with foreign stakeholders, such as governments, communities, and local customers (Francis et al., 2014). Xia et al. (2017) found that, if network partners have engaged in a CBA in a particular country, knowledge and experience in respect of that country will have been generated, and the firm which aims to undertake a future CBA may benefit from this network relationship and perform better by adopting the same strategy in that country.

Specifically, by observing the behaviors of an interlocking partner that has intensively conducted CBAs, an entrepreneur can learn what, and how, to adapt to a new foreign market (Salomon and Wu, 2012). In addition, an interlocking partner with a variety of CBA practices often generates spillovers of information about local markets (Kim and Miner, 2007), which enables the entrepreneur to better understand the acquisition know-how and other details pertaining to a country, including its politics, economy, cultures, and market regulations. Thereby, the firm can become more familiar with the country-specific environment and reduce the liabilities of newness and foreignness.

Interlocking partners also amass social connections, government support, and corporate alliances during the process of CBAs (Daily et al., 2000), and these resources can likewise be delivered and shared through interlocking directors. This increases the firm’s ability to cope with country-specific uncertainty, decreases potential conflicts, and enhances external legitimacy (Zimmerman and Zeitz, 2002). Therefore, by building an interlocking director network with experienced partner companies, entrepreneurs can greatly reduce their psychological barriers, raise their confidence, and increase their ability to cope with the uncertainties in making CBAs.

Furthermore, the knowledge obtained from other companies via interlocking directors is often vivid, complete, and fine grained in relation to their international experience and practices, and such knowledge may not be available from other public sources (Haunschild and Beckman, 1998). We therefore propose:

Hypothesis 1. Through vicarious learning, an entrepreneur can advance its firm’s CBA completion in a country where an interlocking partner has completed an international acquisition.

Different learning approaches through the two types of network connections can influence the effect of knowledge acquisition. Following Cai and Sevilir (2010), we further consider entrepreneur learning from interlocking partners via first-degree versus second-degree connections. As noted above, a first-degree connection involves an interlocking director personally experiencing the CBA practices in the interlocking partner company. Utilizing this type of connection, an entrepreneur can directly observe and learn the international information and experiences of interlocking partners, understand acquisition know-how, and share social resources in a certain target country. Such direct experience is often vivid and can accelerate the firm’s increased familiarity with the issues related to CBAs.

In addition, learning through first-degree connections increases the reliability and completeness of the relevant knowledge acquired from interlocking partners, and helps to establish trust and reciprocity between the two companies, which significantly reduces the risk of opportunistic behaviors and enhances communication efficiency. Moreover, learning from first-degree connections promotes the exchange and transmission of information, knowledge, and resources among companies (Reagans and McEvily, 2003), which maximizes an entrepreneur’s gains from interlocking partners and greatly mitigates their psychological stress in promoting their firm’s CBA completion.

A second-degree connection, on the other hand, involves two interlocking directors who jointly hold positions on the same board of a third company, and entrepreneur learning through such connections indirectly secures international knowledge and experience from interlocking partners, which may not only decrease the efficiency of the information transfer but also compromise the completeness of the required knowledge. Moreover, an interlocking network built on second-degree connections increases the distance between the entrepreneur and the learning objects, so the learning effect may be weaker and it might only play a limited role in lowering entrepreneurs’ psychological stress and helping them to cope with uncertainties in international investments.

However, despite this indirect learning effect, learning from second-degree connections can also provide diversified information and access to a greater number of companies, which increases the degree of information richness compared to that obtained through first-degree connections (Cai and Sevilir, 2010). Entrepreneur learning from interlocking partners based on second-degree connections stimulates the acquisition and accumulation of more extensive international information and knowledge, which enhances an entrepreneur’s understanding of the foreign country and improves the likelihood of completing CBAs.

In sum, the two types of interlocking network connections can have different effects on an entrepreneur’s knowledge acquisition from other companies. Specifically, the CBA experience and management practices of an interlocking partner can be learned in a more reliable and complete way through first-degree connections, while such knowledge and experience might be broader and richer when garnered from second-degree connections. To some extent, both types of connection support the entrepreneur in learning international knowledge from interlocking partners, and lead to a higher probability of completing a CBA in another country. Thus:

Hypothesis 2a. An entrepreneur learning country-specific knowledge via a first-degree connection will promote its firm’s CBA completion.

Hypothesis 2b. An entrepreneur learning country-specific knowledge via a second-degree connection will promote its firm’s CBA completion.

### The Moderating Effect of Knowledge Absorption

Within the context of vicarious learning, externally acquired knowledge is likely to be more effectively absorbed into a firm’s management practices (Giuliani and Bell, 2005). Prior studies have posited that firms with higher absorptive capability are more likely to respond to external uncertainties and perform better in market competition (Escribano et al., 2009). Knowledge absorption requires an individual to better understand, digest, and utilize the acquired knowledge. Notably, Francis and Zheng (2010) have argued that learning from an object with a similar knowledge structure can enhance the ability and effect of knowledge absorption.

An entrepreneurial company possessing a certain knowledge reserve and sharing a similar knowledge base with an interlocking partner can promote knowledge transfer, exchange, and absorption between the two parties (Cohen and Levinthal, 1990). Any knowledge gap may increase the difficulty in understanding and accepting external knowledge learning from other companies, but such a gap can be narrowed if the entrepreneur acquires knowledge from an interlocking partner in the same industry. Within the same industrial environment, two companies will tend to have a high level of knowledge relatedness, which not only promotes knowledge transfer between the two companies but also enables the entrepreneur to experience a better understanding and absorption of CBA knowledge learning from an interlocking partner (Tanriverdi and Venkatraman, 2005; Makri et al., 2010). In addition, the entrepreneur can more easily assimilate the interlocking partner’s experience in CBA decision-making, evaluation of a target company, and dealing with local key stakeholders, and such experience can be effectively applied into their firm’s own CBA practices.

Therefore, building interlocking connections with companies in the same industry significantly enhances an entrepreneur’s capability of absorbing international knowledge from interlocking partners. In doing so, entrepreneurs increase their confidence in dealing with mental stress and with overcoming uncertainties, which better promotes CBA completion and their firm’s international growth. Accordingly:

Hypothesis 3. The positive relationship theorized in Hypothesis 1 will be stronger when the entrepreneur enhances knowledge absorption through making a connection with an interlocking partner in the same industry.

### The Moderating Effect of Knowledge Application

The outcomes of vicarious learning also rely on the process of knowledge application, which itself emphasizes the extent to which the acquired knowledge can be utilized and affect a firm’s strategy and performance. In relation to a firm’s CBA investments, the application of international knowledge learning from an interlocking partner will be influenced by the institutional environment in the target country.

Making CBAs in a target country with a high degree of openness reduces an entrepreneur’s need for international knowledge learning from other companies. When their firm is undertaking a CBA in a highly open country, an entrepreneur can easily obtain information and knowledge from the formal, transparent, and fair transaction market therein, which means that they can become familiar with the country’s institutions and culture in other public ways, such as social media, rather than drawing on knowledge learning from interlocking partners. Equally, an entrepreneur may decrease psychological distance and be able to better predict potential risks and costs in a highly open country, thus limiting the application of acquired international experience and know-how to their firm’s CBA investments.

Conversely, for a firm undertaking CBAs in a target country with a low degree of openness, the entrepreneur must rely on knowledge and experience learning from interlocking partners to reduce uncertainty and potential risk. Also, entrepreneurs often find it difficult to acquire information and accumulate social resources in a less transparent country, which increases the need for international knowledge acquired from experienced companies and strengthens the application of learning experience to their firms’ CBA strategy. This issue is of particular importance for most entrepreneurs with less or no international experience (Shimizu et al., 2004). We thus propose:

Hypothesis 4. The positive relationship theorized in Hypothesis 1 will be stronger when the entrepreneur enhances knowledge application through internationally investing in a country with a lower degree of openness.

**FIGURE 2:**
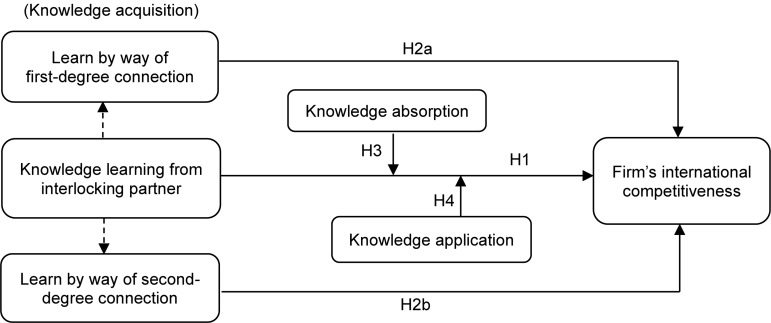
Research theoretical framework.

As an overview, Figure 2 illustrates our study’s theoretical framework and shows the logic relationships among the main variables.

## Research Methods

We selected Chinese companies that had conducted CBAs between 2008 and 2017 as our research sample. The Chinese sample is advantageous and appropriate for several reasons. First, there is rapid growth of new ventures in China, and most of them are eyeing overseas markets to improve their firm’s competitiveness (Wang et al., 2012; Alon et al., 2018). Second, Chinese companies stay in the initial process of internationalization, thus entrepreneurs face much pressure and uncertainties to deal with liabilities of newness and foreignness (Child and Rodrigues, 2005; Peng, 2005; Rugman et al., 2016). Last, an interlocking director network is intensive and widespread among Chinese companies (Peng and Luo, 2000; Li et al., 2013), which exerts a significant influence on the firm’s decision and performance.

Financial data and CBA information come from the Wind database and CSMAR databases, both of which are widely used in Chinese acquisition and management research (Giannetti et al., 2015; Greve and Zhang, 2017). National-level data were collected from the World Bank database and the United Nations Statistical Database. After excluding samples with missing data and inappropriate CBA transactions, we obtained 971 observations encompassing 540 companies and 55 target countries/regions with CBAs.

### Measurements

#### Dependent Variable

Cross-border acquisitions completion was represented by a dummy variable coded as “1” if a deal was completed, and “0” otherwise (Li et al., 2017).

#### Independent Variables

We measured vicarious learning knowledge (VLK) by calculating the number of CBAs conducted by the interlocking partner in the past three years in the same target country as that targeted by the focal firm’s CBA. The measurement of knowledge learning through a first-degree connection (Tier 1_VLK) and a second-degree connection (Tier 2_VLK) was calculated in the same way. We lagged all of these variables by one year (Hair et al., 2006).

#### Moderating Variables

Knowledge absorption was measured with reference to the industry relatedness between the focal firm and the interlocking partner company, a dummy variable set to equal “1” if the industry code was the same between the firm and the interlocking partner, and “0” otherwise. The industry codes followed the Guidelines of Industry Classification of Listed Companies issued by the China Securities Regulatory Commission (2012).

Regarding knowledge application, we measured target country openness using the “trade freedom” score component of the Index of Economic Freedom published by the Heritage Foundation, which reflects the intensity of foreign trade. Scores range from 0 to 100, and a country ranked as having high trade freedom tends to have a greater degree of openness to the outside world and features more developed institutional systems (Guttmann and Richards, 2006).

#### Control Variables

Following previous research (Li et al., 2017), we controlled for firm-level attributes. In consideration of the effect of a firm’s own international experience (Lee and Caves, 1998; Clarke et al., 2013), we calculated the number of CBA attempts made in the past three years (prior experience). In addition, we controlled for the deal attributes of same industry acquisition and deal size, following the research of Li et al. (2018), with deal size measured by the logarithm of the total announced value of the focal transaction. Given that larger firms are more likely to undertake international investments as they possess stronger financing abilities (Jovanovic and Braguinsky, 2004; Cleary, 2006), we also measured firm size, using the logarithm of the firm’s total assets. As a firm’s performance may influence the opportunity for foreign investment initiatives (Bushman et al., 2005), we controlled for Tobin’s q and return on assets (ROA). Additionally, we controlled for state ownership, because of its impact on a firm’s international investment (Cui et al., 2015), and measured it using the ratio of the firm’s state-owned shares.

Furthermore, we controlled for country-level attributes (Gubbi et al., 2010; Nicholson and Salaber, 2013) through reference to the target country’s economic development, such as its gross domestic product (GDP), GDP growth rate, and Economic Freedom Index ranking (EFI). In addition, we controlled for the institutional distance and cultural distance between the home country and the target country, with data for a country’s institutional quality collected from the World Governance Index and that for the country’s cultural data taken from Hofstede’s cultural indices. These variables were measured using the methods recommended by Kogut and Singhm (1988).

## Results

### Descriptive Statistics

Table 1 presents the descriptive statistics for all the variables. On average, only 55% of firms completed a CBA, suggesting a lower success rate for CBAs conducted by Chinese firms. Regarding the vicarious learning through which entrepreneurs accumulated CBA knowledge from interlocking partners, about twice as much knowledge was obtained through second-degree connections as through first-degree connections, suggesting that a greater number of companies being connected offers enhanced opportunities to obtain international knowledge and information via second-degree connections. As regards knowledge absorption, about 17% of companies were in the same industry as their interlocking partners. For knowledge application, most of the companies undertook CBAs in a highly open country, with an average trade freedom index value of 85.96. Table 2 presents the correlation coefficients among the variables. It can be seen that the value of each variable is under 0.6, indicating that multicollinearity was not a significant problem in the regression analyses.

**TABLE 1:** Descriptive statistics.

**Variable**	**Mean**	**SD**	**Min.**	**Max.**
CBA completion	0.55	0.49	0.00	1.00
VLK	0.08	0.31	0.00	3.00
Tier 1_VLK	0.02	0.16	0.00	2.00
Tier 2_VLK	0.05	0.25	0.00	2.00
Knowledge absorption	0.17	0.37	0.00	1.00
Knowledge application	85.96	5.00	44.20	95.00
Prior experience	0.36	0.77	0.00	5.00
Same industry acquisition	0.47	0.50	0.00	1.00
Deal size	9.51	2.34	0.13	16.43
Firm size	22.69	1.93	18.61	30.66
Tobin’s q	2.62	2.86	0.07	26.04
ROA	0.05	0.06	−0.52	0.67
State ownership	0.02	0.10	0.00	0.86
GDP	7.61	1.52	2.67	9.91
GDP growth rate	2.33	1.74	−8.27	25.01
EFI	75.60	8.58	44.55	90.14
Institutional distance	3.33	1.27	0.00	5.98
Cultural distance	2.58	1.42	0.00	8.33

**TABLE 2:** Pearson correlation coefficients.

	**1**	**2**	**3**	**4**	**5**	**6**	**7**	**8**	**9**	**10**	**11**	**12**	**13**	**14**	**15**	**16**	**17**
(1) CBA completion	1																
(2) VLK	0.08***	1															
(3) Tier 1_VLK	0.08**	0.48***	1														
(4) Tier 2_VLK	0.05*	0.54***	0.06*	1													
(5) Knowledge absorption	0.03	0.36***	0.20***	0.31***	1												
(6) Knowledge application	0.06**	0.06**	0.03	0.06*	0.03	1											
(7) Prior experience	0.01	0.04	0.01	0.04	0.07**	0.01	1										
(8) Same industry acquisition	0.08***	0.01	0.01	0.01	0.06*	–0.03	0.05*	1									
(9) Deal size	0.07**	0.07**	0.06**	0.05	–0.01	–0.04	0.16***	0.07**	1								
(10) Firm size	0.01	0.02	0.01	0.02	0.02	−0.07**	0.28***	0.13***	0.45***	1							
(11) Tobin’s q	−0.05*	−0.06**	–0.01	−0.07**	−0.07**	0.03	−0.10***	–0.02	0.16***	−0.46***	1						
(12) ROA	–0.00	–0.01	–0.03	0.01	0.02	0.05	–0.01	0.03	–0.04	−0.09***	0.29***	1					
(13) State ownership	0.04	–0.04	–0.01	–0.04	−0.08**	−0.07**	0.10***	0.12***	0.36***	0.61***	−0.27**	−0.10**	1				
(14) GDP	−0.06*	0.13***	0.04	0.13***	0.09***	−0.11***	0.03	0.03	–0.04	−0.10***	0.07**	0.06*	−0.09***	1			
(15) GDP growth rate	0.02	0.01	0.01	0.00	–0.02	−0.13***	–0.04	–0.04	0.01	–0.01	0.01	–0.04	–0.04	−0.17***	1		
(16) EFI	0.01	0.07**	0.06**	0.04	–0.04	0.52***	–0.04	−0.14***	0.07**	0.01	0.01	0.02	0.03	−0.24***	0.03	1	
(17) Institutional distance	0.02	0.01	0.03	–0.01	–0.03	0.58***	–0.04	−0.05*	0.03	–0.01	0.01	0.06*	0.10***	−0.18***	−0.17***	0.67***	1
(18) Cultural distance	–0.04	0.03	0.02	0.02	0.10***	–0.02	0.06*	0.05*	0.02	–0.05	0.05	0.08***	0.01	0.40***	−0.11***	−0.23***	0.15***

*p < 0.1, **p < 0.05, ***p < 0.01.

**FIGURE 3:**
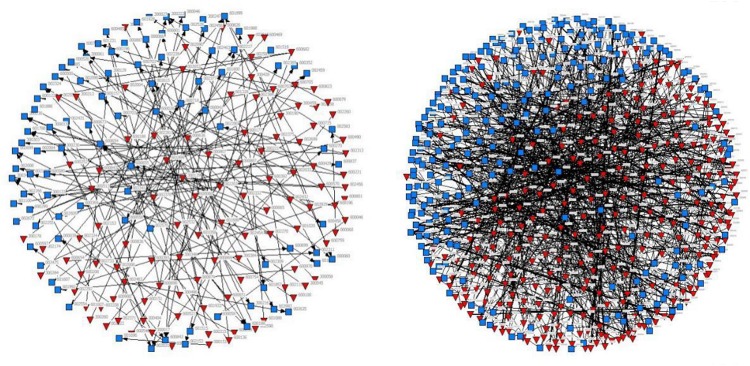
Comparison of two types of interlocking networks. The left is network shaped by first-degree connections; the right network shaped by second-degree connections.

Figure 3 compares the two approaches to learning through first-degree connections (left) and second-degree connections (right). In the network diagram, the inverted triangles represent the entrepreneurs’ firms and the squares represent the interlocking partners, with only interlocking partners with CBA experience being included. The line represents the number of interlocking directors connecting the two companies—that is, the strength of the interlocking connection. It can be clearly seen that the network shaped by first-degree connections is not as intensive as the one shaped by second-degree connections—that is, the number of companies involved is less than that in the network of second-degree connections. The learning approach utilizing second-degree connections reaches out to more companies with CBA experience, which can provide more diversified and abundant CBA information and knowledge for the entrepreneurs. This finding further supports our theorizing on the different roles of the two types of approaches to learning; that is, learning through first-degree connections directly accesses the CBA experience of interlocking partners, while learning through second-degree connections does so indirectly, but can connect a greater number of interlocking partners through which to acquire CBA knowledge.

### Regression Results

We adopted a logistic model to test our hypotheses with the dependent variable as a dummy variable, following Li et al. (2018) and Dikova et al. (2010). The regression results are shown in Table 3. Model 1 is the baseline model with all the control variables. The results show that a CBA with large deal size and between an acquiring firm and a target company in the same industry is more likely to be completed. However, a firm of a large size or with a high value of Tobin’s q has less likelihood of completing a CBA.

**TABLE 3:** Results of vicarious learning knowledge and firms’ international investment.

**Variables**	**Model 1**	**Model 2**	**Model 3**	**Model 4**	**Model 5**	**Model 6**
VLK		0.38***			0.02	10.46*
		(0.13)			(0.21)	(6.08)
Tier 1_VLK			0.73**			
			(0.29)			
Tier 2_VLK				0.28*		
				(0.16)		
Knowledge absorption					–0.04	
					(0.12)	
Knowledge absorption × VLK					0.61**	
					(0.29)	
Knowledge application						0.03***
						(0.01)
Knowledge application × VLK						−0.11*
						(0.06)
Prior experience	0.04	0.04	0.03	0.04	0.04	0.05
	(0.11)	(0.11)	(0.11)	(0.11)	(0.11)	(0.11)
Same industry acquisition	0.22***	0.21***	0.21***	0.22***	0.21**	0.20**
	(0.08)	(0.08)	(0.08)	(0.08)	(0.08)	(0.08)
Deal size	0.05***	0.04**	0.04**	0.05**	0.04**	0.05***
	(0.01)	(0.01)	(0.01)	(0.01)	(0.02)	(0.02)
Firm size	−0.06**	−0.06**	−0.06**	−0.06**	−0.06**	−0.06**
	(0.03)	(0.03)	(0.03)	(0.03)	(0.03)	(0.03)
Tobin’s q	−0.03**	−0.03*	−0.03**	−0.03*	−0.03*	−0.03*
	(0.01)	(0.01)	(0.01)	(0.01)	(0.01)	(0.01)
ROA	0.42	0.41	0.47	0.39	0.37	0.39
	(0.63)	(0.63)	(0.63)	(0.63)	(0.64)	(0.63)
State ownership	0.18	0.23	0.19	0.21	0.21	0.28
	(0.27)	(0.27)	(0.27)	(0.27)	(0.27)	(0.27)
GDP	–0.02	–0.04	–0.03	–0.03	–0.04	–0.04
	(0.03)	(0.03)	(0.03)	(0.03)	(0.03)	(0.03)
GDP growth rate	0.01	0.01	0.01	0.01	0.01	0.02
	(0.02)	(0.02)	(0.02)	(0.02)	(0.02)	(0.02)
EFI	–0.01	–0.01	–0.01	–0.01	–0.01	−0.01**
	(0.01)	(0.01)	(0.01)	(0.01)	(0.01)	(0.01)
Institutional distance	0.06	0.06	0.06	0.06	0.05	0.03
	(0.05)	(0.05)	(0.05)	(0.05)	(0.05)	(0.05)
Cultural distance	–0.04	–0.04	–0.05	–0.04	–0.04	–0.05
	(0.03)	(0.03)	(0.03)	(0.03)	(0.03)	(0.03)
Constant	1.69**	1.93**	1.80**	1.81**	1.90**	–0.17
	(0.84)	(0.85)	(0.85)	(0.85)	(0.85)	(1.14)
Observations	971	971	971	971	971	971

Numbers in parentheses are standard errors. *p < 0.1, **p < 0.05, ***p < 0.01.

In Model 2, international knowledge learned from interlocking partners significantly advances a firm’s likelihood of a CBA completion, which supports Hypothesis 1. This result suggests that, for entrepreneurs making international investments, vicarious learning is an effective way to reduce their psychological stress and accumulate international knowledge from other experienced companies with which to promote their firms’ international growth. Models 3 and 4 added two variables based on the approaches to learning through first-degree and second-degree connections. The results show that international knowledge obtained through both types of interlocking connections significantly enhances a firm’s likelihood of CBA completion. However, the coefficient of learning by way of first-degree connections (0.73) is higher than that for second-degree connections (0.28), indicating that direct knowledge acquisition through a first-degree connection is more reliable and has a greater impact on a firm’s international strategy than indirect knowledge acquisition through a second-degree connection, despite the possibility that second-degree connections can reach out to a greater number of companies and more diversified information. Hypotheses 2a and 2b are therefore supported.

Model 5 represents the moderating effect of knowledge absorption. Herein, we examined industry similarity between the focal firm and the interlocking partner. It can be seen that the two companies being in the same industry has a positive moderating effect (0.61, *p* < 0.05) on the influence of an interlocking partner’s knowledge on CBA completion, which supports Hypothesis 3. This result is consistent with Escribano et al.’s (2009) finding that firms with high absorptive capability can derive greater benefits from external knowledge and that this positively affects performance. Model 6 further depicts the moderating role of knowledge application. We posited that the effect of knowledge application would be weaker when the entrepreneur undertook a CBA in a highly open country. Model 6 shows that a target country’s trade freedom has a negative moderating role (−0.11, *p* < 0.10) in the relationship between the interlocking partner’s knowledge and CBA completion, which supports our argument that making international investments in a highly open country will bring about a substitute effect, and thus reduce the entrepreneur’s need for knowledge and experience learning from interlocking partners. Accordingly, Hypothesis 4 is also supported.

## Discussion

Economic developments in emerging markets have facilitated the emergence of many new companies in recent years, but the concomitant increases in competition and uncertainty in the external environment have also been found to negatively affect entrepreneurs. The psychology of entrepreneurs has attracted extensive academic attention (Gorgievski and Stephan, 2016), and, drawing on the learning psychology perspective, the present paper focused on firms’ international investments and explored a vicarious learning approach for entrepreneurs accumulating international knowledge and experience through learning from interlocking director network partners. We described vicarious learning as being obtainable through both first-degree connections and second-degree connections, and empirically examined how knowledge absorption and knowledge application moderate the effect of an interlocking partner’s knowledge on the entrepreneurial firm’s international development. Based on a study of CBAs conducted by Chinese firms, our research revealed some important findings.

First, this paper contributes to the learning psychology research by elucidating a vicarious learning approach for entrepreneurs to reduce stress by learning from experienced companies based on an interlocking director network. We emphasized that constructing an interlocking director network is an effective way for entrepreneurs—especially those with little or no international experience—to learn about the decision-making, behaviors, and experiences of their interlocking partners and thereby accelerate their own international knowledge accumulation, which in turn enhances their ability and confidence in dealing with external uncertainty, and improves their firms’ international competitiveness. Second, as noted, this paper contributes to prior studies by differentiating two vicarious learning approaches involving first-degree versus second-degree connections (Cai and Sevilir, 2010). We found that entrepreneurs learning from first-degree connections could obtain direct experience from their interlocking partners and enhance the efficiency of information transfer, which was found to have a more significant positive effect on entrepreneurs and their firms’ international strategy. On the other hand, learning via second-degree connections was shown to allow entrepreneurs to connect with a greater number of companies and obtain more diversified information, but this approach’s indirect learning effect may have a limited role in respect of the entrepreneur firm’s international strategy.

In common with extant learning research, our paper also explored three stages of the vicarious learning process (i.e., knowledge acquisition, knowledge absorption, knowledge application), and examined the moderating effects of knowledge absorption and knowledge application. We found that companies with similar knowledge structures could better understand and absorb the knowledge learning from their interlocking partners. Specifically, being in the same industry was found to positively moderate the effect of acquired international knowledge on a firm’s CBA completion. This finding is consistent with Francis and Zheng’s (2010) assertion that a small knowledge gap between two actors can promote the effect of knowledge absorption. Additionally, regarding the moderating role of knowledge application, our research found that an entrepreneur undertaking a CBA in a highly open country can acquire information and resources from a transparent and open transaction market, and thereby reduce their dependence on learning international experience from interlocking partners. That is, target country openness weakens an entrepreneur’s application of acquired international knowledge and thus plays a negative moderating role in this context.

### Theoretical and Practical Implications

Overall, our study advances some significant theoretical implications. We have provided novel insights for entrepreneurs wanting to improve their firms’ international competitiveness by introducing an approach to vicarious learning based on interlocking directors, which should reduce entrepreneurs’ psychological stress and help them better cope with external uncertainty. Additionally, we have distinguished between two types of interlocking network (based on first-degree connections versus second-degree connections) in the context of China. As interlocking director networks are common and tend to be an important source of knowledge transfer, exploring the influence of an interlocking partner’s international knowledge on a focal firm’s international investment is of theoretical significance. In addition, through our exploration of the processes of knowledge absorption and knowledge application and their moderating effects on the external knowledge acquisition, we have enriched the current research concerning vicarious learning.

With respect to the practical implications of our findings, we first suggest that entrepreneurs should pay attention to the key role of interlocking directors in obtaining the necessary knowledge for international strategies. Especially when companies have less experience in global investments, they can learn the applicable international experience from their interlocking partners through establishing connections via interlocking directors, and improve the likelihood of success in international investments. Second, when seeking knowledge and experience with respect to international investments, entrepreneurs should consider a strong relationship with a first-degree connection, which can provide high-quality information, and then consider learning through second-degree connections, which will provide rich and diverse information. Last, possessing heightened capabilities of knowledge absorption and application will enable entrepreneurs to benefit more from knowledge acquired from other companies. Making interlocking connections with companies in the same industry as their own can enhance the ability of knowledge absorption and thereby positively influence a firm’s strategy and development.

In addition, ourpaper also provides some promising directions for further research. For example, scholars could examine entrepreneurs’ vicarious learning through other channels, such ascompany alliances and corporate business relationships. Also, further research can explore more specific and appropriate measurements of knowledge absorption and application, such as which characteristics of entrepreneurs orcorporate governance enhance the capability for absorbing external knowledge.

## Data Availability Statement

The datasets for this article are not publicly available, because they contain entrepreneurs’ personal information. Request to access the datasets should be directed to the corresponding author.

## Ethics Statement

The studies involving human participants were reviewed and approved by the Zhejiang Gongshang University. The patients/participants provided their written informed consent to participate in this study.

## Author Contributions

ZX and WH analyzed the data and wrote the manuscript. RL and JW conceived the idea of the manuscript and designed the research. ZX and JW revised the manuscript. LM provided constructive suggestions to improve the research. All authors have read and approved the final manuscript.

## Conflict of Interest

The authors declare that the research was conducted in the absence of any commercial or financial relationships that could be construed as a potential conflict of interest.
